# Comparison of the replication and neutralization of different SARS‐CoV‐2 Omicron subvariants in vitro

**DOI:** 10.1002/ame2.12302

**Published:** 2023-02-20

**Authors:** Yaqing Zhang, Qi Lv, Feifei Qi, Fengdi Li, Ran Deng, Xujian Liang, Mingya Liu, Yiwei Yan, Linlin Bao

**Affiliations:** ^1^ Beijing Key Laboratory for Animal Models of Emerging and Reemerging Infectious Diseases, NHC Key Laboratory of Human Disease Comparative Medicine Institute of Laboratory Animal Sciences, Chinese Academy of Medical Sciences & Peking Union Medical College Beijing China; ^2^ National Center of Technology Innovation for Animal Model Beijing China

**Keywords:** cross‐neutralize, Omicron, replication, SARS‐CoV‐2

## Abstract

**Background:**

New Omicron subvariants are emerging rapidly from BA.1 to BA.4 and BA.5. Their pathogenicity has changed from that of wild‐type (WH‐09) and Omicron variants have over time become globally dominant. The spike proteins of BA.4 and BA.5 that serve as the target for vaccine‐induced neutralizing antibodies have also changed compared to the previous subvariants, which is likely to cause immune escape and the reduction of the protective effect of the vaccine. Our study addresses the above issues and provides a basis for formulating relevant prevention and control strategies.

**Methods:**

We collected cellular supernatant and cell lysates and measured the viral titers, viral RNA loads, and E subgenomic RNA (E sgRNA) loads in different Omicron subvariants grown in Vero E6 cells, using WH‐09 and Delta variants as a reference. Additionally, we evaluated the in vitro neutralizing activity of different Omicron subvariants and compared it to the WH‐09 and Delta variants using macaque sera with different types of immunity.

**Results:**

As the SARS‐CoV‐2 evolved into Omicron BA.1, the replication ability in vitro began to decrease. Then with the emergence of new subvariants, the replication ability gradually recovered and became stable in the BA.4 and BA.5 subvariants. In WH‐09‐inactivated vaccine sera, geometric mean titers of neutralization antibodies against different Omicron subvariants declined by 3.7~15.4‐fold compared to those against WH‐09. In Delta‐inactivated vaccine sera, geometric mean titers of neutralization antibodies against Omicron subvariants declined by 3.1~7.4‐fold compared to those against Delta.

**Conclusion:**

According to the findings of this research, the replication efficiency of all Omicron subvariants declined compared with WH‐09 and Delta variants, and was lower in BA.1 than in other Omicron subvariants. After two doses of inactivated (WH‐09 or Delta) vaccine, cross‐neutralizing activities against various Omicron subvariants were seen despite a decline in neutralizing titers.

## Graphical abstract

Different Omicron subvariants were inoculated into Vero E6 cells. The cell supernatants and lysate products were collected at different time points. The supernatants were used to determine the viral loads and titers, and the cell lysate products were used to determine the viral E subgenome. Seras from macaques vaccinated with different inactivated vaccines (WH‐09 or Delta) were also collected at 14 days post infection for determination of cross‐neutralizing antibody activity. WH‐09 and Delta were used as controls.

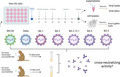

## 1 INTRODUCTION

The Omicron BA.1 (B.1.1.529) variant of SARS‐CoV‐2 rapidly dominated the coronavirus disease‐2019 (COVID‐19) pandemic within weeks of its emergence. BA.1 underwent considerable changes, including changes to 39 amino acid residues in the S protein^1^ associated with increased immune escape and viral spread. BA.2 then replaced previous subvariants BA.1 and BA.1.1 and began to spread rapidly. It had increased S371F (S371L for BA.1), T376A, D405N, R408S, and G446S^−^ mutations compared to BA.1. Following on from BA.2, the BA.2.12.1 subvariant had increased L452Q and S704L mutations. BA.2.12.1 increased significantly in the United States after its appearance there, with BA.2.12.1 infections accounting for 43% of all infections as of early May. BA.4 and BA.5, which added two more alterations, L452R, and F486V, rapidly became dominant in the USA and on the rise worldwide since their emergence in May, accounting for, respectively, 85.5% and 7.7% of infections.^2^ All of the above new subvariants showed different degrees of increased infectivity and enhanced immune escape ability compared with BA.1.^3^, ^4^ Recent research has demonstrated that BA.4 and BA.5 are neutralized by vaccine‐induced antibodies less effectively than BA.1 and BA.2.^5^

The development of new Omicron subvariants and the increase in mutation sites have led to changes in the pathogenicity and antibody neutralization capability of the virus, and the degree of protection offered by currently available vaccines against the new mutant strains is an alarming problem. There is thus a very immediate and pressing need to research the in vitro replication and neutralization of these new subvariants of Omicron. We therefore investigated the differences in viral loads, titers, and subgenomic loads between several major subvariants of Omicron and previous strains (WH‐09 and Delta variants) during their growth and replication in vitro. We also determined the in vitro neutralizing activity for these new subvariants of Omicron. We compared this to the WH‐09 and the Delta variants in sera from macaques that had received different two‐dose vaccines.

## 2 METHODS

### 2.1 Cells and viruses

Vero E6 cells were cultured in Dulbecco's modified Eagle's medium (DMEM, Invitrogen) supplemented with 10% inactivated fetal bovine serum (FBS), 100 IU/mL penicillin, and 100 μg/mL streptomycin at 37°C and 5% CO_2_ without mycoplasma contamination. SARS‐CoV‐2 WH‐09 (Genbank: MT093631), Delta (Genbank: OM061695), BA.1 (Genbank: OM095411), BA.2 (Genbank: OP678015), BA.2.12.1 (Genbank: OP678014), BA.4 (Genbank: OP678017), and BA.5 (Genbank: OP678016) were provided by the Institute of Laboratory Animals Sciences, CAMS & PUMC; BA.2, BA.2.12.1, BA.4 and BA.5 were collected by Guangdong Provincial Center for Disease Control and Prevention, and the virus was amplified using Vero E6 cells. Virus titers were established using 50% tissue‐culture infectious doses (TCID_50_). SARS‐CoV‐2 experiments were performed in the Institute's BSL‐3 laboratory according to the appropriate safety and security regulations.

### 2.2 Viral titer

The virus was diluted 10‐fold, inoculated into Vero E6 cells, and incubated at 37°C. After 1 hour, the diluted solution was added to 200 μl DMEM containing 2% FBS and the same amounts of penicillin and streptomycin as described for cell culture, and then incubated at 37°C, 5%CO_2_. The cytopathic effect was examined under the microscope after 3 days, and the TCID_50_ was estimated using the Reed‐Muench method.

### 2.3 Virus growth and replication assay in vitro

Vero E6 cells were inoculated onto 24‐well cell plates. When the cell density reached 5 × 10^5^ cells/well, the original culture medium was discarded and the cells were incubated for 1 hour in a 20TCID_50_ virus culture solution per well. The virus culture solution was then discarded and the cells were incubated with 1 ml of DMEM with 2% FBS and the same amounts of penicillin and streptomycin as described for cell culture to continue the culture. The cell supernatants were collected at time 0 (directly after replacement with cell culture medium), and 12, 24, 48 and 72 h post infection to test the viral loads and titers, and cell lysates collected at the same time points were used for viral E subgenome RNA detection. Three replicates were performed for each time point.

### 2.4 RNA extraction and RT–qPCR

The RNeasy Mini Kit (Qiagen) was utilized to extract RNA from cell supernatants and lysates. For evaluating viral RNA levels, the PrimerScript RT Reagent Kit (TaKaRa) was used to perform the first reverse transcription following the manufacturer's instructions. RT–qPCR reactions were performed using the PowerUp SYBG Green Master Mix Kit (Applied Biosystems) and the following cycling protocol: 50°C for 2 minutes, 95°C for 2 min, then 40 cycles of 95°C for 15 s and 60°C for 30 s, followed by 95°C for 15 s, 60°C for 1 minute, and 95°C for 45 s. The following primer sequences were used for RT–qPCR to target the envelope (E) gene of SARS‐CoV‐2:

forward: 5′‐TCGTTTCGGAAGAGACAGGT‐3′,

reverse: 5′‐GCGCAGTAAGGATGGCTAGT‐3′.

We assessed viral E subgenomic mRNA (sgRNA) loads using a one‐step RT–qPCR based on previously described methods.^6^, ^7^ Briefly, the primer sequences consist of the following:

forward: 5′CGATCTCTTGTAGATCTGTTCTCE3′;

reverse: 5′ATATTGCAGCAGTACGCACACA3′;

probe: 5′ FAM‐ACACTAGCCATCCTTACTGCGCTTCG‐BHQ1 3′.

### 2.5 Immune experiments using macaques

Six macaques (4–7 years old) were randomly divided into two groups, one of which was immunized intramuscularly with two 6 μg doses of inactivated vaccines against WH‐09 given 28 days apart. The three macaques in the other group were vaccinated intramuscularly with inactivated vaccines against Delta at the same dose and time interval as in the first group. All serum samples were collected at 14 days after the second immunization for neutralizing antibody determination.

### 2.6 Neutralizing antibody assay

Using cytopathic effect (CPE) testing, the existence of neutralizing antibodies was determined. In detail, after inactivation at 56°C for 30 minutes and serial 2‐fold gradient dilutions, sera samples were incubated at 37°C with 100 TCID_50_ SARS‐CoV‐2. After 1 h, the liquid mixtures were added to Vero‐E6 cells in 96‐well plates, the cells were cultured at 37°C for 3–4 days, and then the cytopathic effects were observed, and the serum dilution that protected 50% of the cells from infection was determined.^8^

### 2.7 Statistical analysis

All data were analyzed using GraphPad Prism 8.0 (GraphPad Software, Inc.) software, and a two‐tailed unpaired Student's test was used to compare results between groups. The statistical significance thresholds were chosen to be *p* < 0.05 (*), *p* < 0.01 (**).

## 3 RESULT

### 3.1 Growth and replication of different SARS‐CoV‐2 strains in Vero E6 cells

**FIGURE 1:**
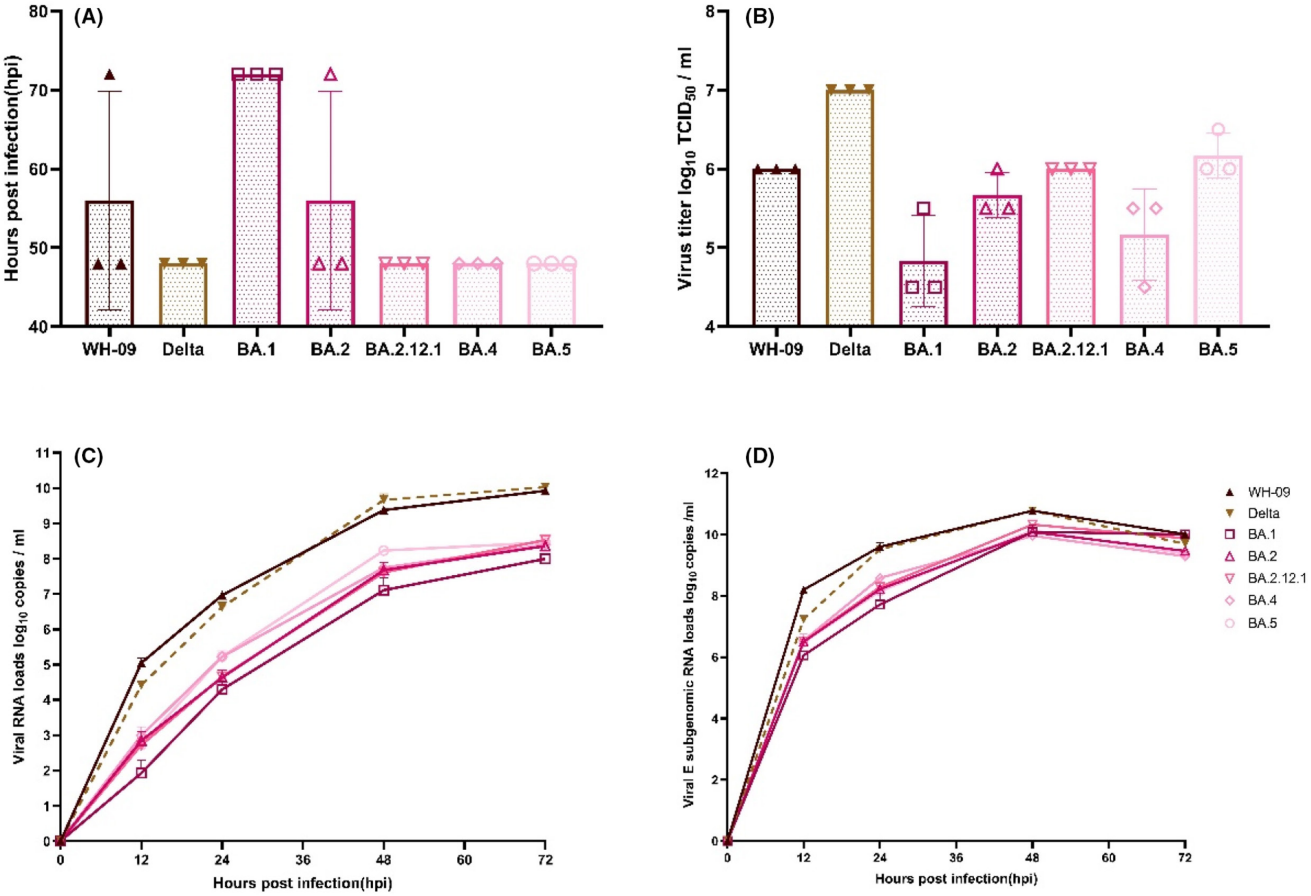
Viral replication of different SARS‐CoV‐2 mutant strains in Vero E6. Cells were infected at 20 TCID_50_ for 72 h, and culture supernatant was collected at the indicated time points to quantify when the virus titers reach a peak (A), distribution of peak titers of the viruses in the supernatants (B) and viral RNA loads (C). Cell lysates were collected to quantify viral E subgenomic RNA loads by RT‐PCR (D); *n* = 3)

We used the Vero E6 cell culture model to analyze the replication kinetics of the different SARS‐CoV‐2 Omicron subvariants BA.1, BA.2, BA.2.12.1, BA.4, and BA.5. For comparison, we also included the WH‐09 and Delta variants. After cells were infected with different strains at 20TCID_50_, we measured the titers of virus particles in the supernatant collected at different time points. The results showed that at 12 h post infection (hpi), the average titers of WH‐09, Delta, BA.2, and BA.2.12.1 were 10^1.5^, 10^1.7^, 10^1.4^, 10^1.4^ TCID_50_/ml, respectively; the titers of BA.1, BA.4 and BA.5 could not be measured (Figure S1). Regarding the time to reach peak viral titer, the Delta strain, and BA.2.12.1, BA.4, and BA.5 mutant strains reached peak replication most rapidly, with a peak time of 48 h. WH‐09 and BA.2 mutants peaked at 56 h after inoculation of cells, and BA.1 peaked at 72 h (Figure 1A). The average peak titers of WH‐09, Delta, BA.1, BA.2, BA.2.12.1, BA.4, and BA.5 mutant strains were 10^6.0^, 10^7.0^, 10^5.1^, 10^5.7^, 10^6.0^, 10^5.3^, 10^6.2^ TCID_50_/ml, respectively (Figure 1B). Compared with WH‐09, the peak titer of the Delta variant increased significantly while that of the Omicron BA.1 strain decreased significantly; the peak titers of BA.2 and BA.2.12.1 showed an increasing trend. The peak titers of the other Omicron subvariants BA.4 and BA.5 showed an increasing trend compared with BA.1.

**FIGURE 2:**
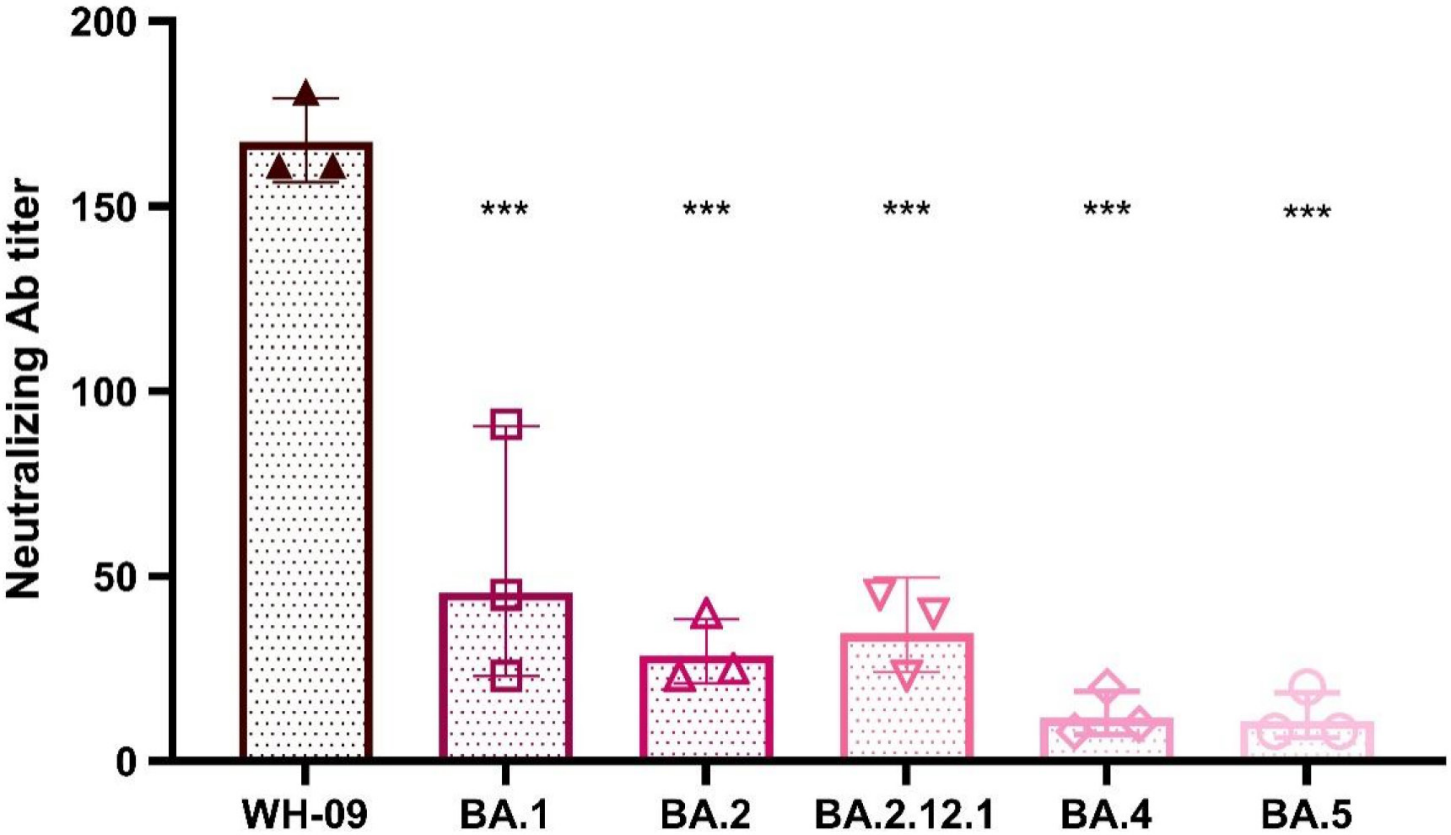
Neutralizing antibody titers against different viruses in sera of macaques after two doses of inactivated vaccine (WH‐09). The error bar shows the standard deviation of three separate experiments.

We then measured the viral loads of the cell supernatants. The viral loads of the WH‐09 and Delta strains exhibited similar amplification efficiencies, and the viral loads of all Omicron subvariants were lower than those prevalent earlier in the WH‐09 and Delta strains. The viral loads of BA.1 were lower than those of other Omicron subvariants. We collected the cell supernatant after 12 h of viral replication. The viral loads of WH‐09 and Delta were 10^5.1^ and 10^4.4^ copies/ml. The viral loads of BA.1 were much lower than those of WH‐09 and Delta, decreasing by 1140.1‐fold compared with WH‐09 and 265.8‐fold compared with Delta. The other Omicron subvariants decreased 107.3 to 205.4‐fold compared with WH‐09 and 25.0 to 47.9‐fold compared with Delta. At 72 hours post infection, all viral loads reached a maximum. The maximum loads of WH‐09 and Delta were 10^9.9^ and 10^10.0^ copies/ml, respectively. The loads of all Omicron subvariants were highly significantly different from both WH‐09 and Delta variants, with BA.1 (10^8.0^ copies/ml) showing an 81.8‐fold reduction compared to WH‐09 and a 102.9‐fold reduction compared to Delta. The remaining Omicron subvariants showed load reductions ranging from 24.9‐ to 36.2‐fold compared with WH‐09 and 31.3‐ to 45.5‐fold compared with the Delta variant (Figure 1C).

The subgenomic results from cell lysates showed that the viral E sgRNA growth rates of all SARS‐CoV‐2 strains were the fastest within 12 hours post infection. At 12 hpi, the viral E subgenome RNA loads of WH‐09 and Delta were 10^8.2^ and 10^7.2^ copies/ml, respectively. The E sgRNA loads of BA.1 were the lowest at 10^6.1^ copies/ml, a 135.3‐fold decrease compared to WH‐09, and the other Omicron subvariants decreased 43.0‐ to 49.8‐fold compared to WH‐09, with a range of 10^6.5^–10^6.6^ copies/ml. The E sgRNA loads of all strains peaked at 48 hpi. The peak E sgRNA loads of both WH‐09 and Delta were 10^10.8^ copies/ml, and the E sgRNA loads of the Omicron subvariants ranged from 10^10.0^ to 10^10.3^ copies/ml (Figure 1D).

### 3.2 Cross‐neutralizing antibodies elicited in sera from macaques immunized with two doses of inactivated vaccines (WH‐09)

We compared the in vitro neutralizing activity of five Omicron subvariants to that of the WH‐09 strain in the serum of macaques vaccinated with two doses of inactivated vaccines (WH‐09). Among all samples, the reduction in the neutralization of Omicron compared with the WH‐09 was up to 15.4‐fold. The sera showed varying degrees of decrease in neutralizing ability against different Omicron subvariants, with 3.7‐fold, 5.9‐fold, and 4.8‐fold reductions in neutralizing activity against BA.1, BA.2, and BA.2.12.1, but 14.3‐fold and 15.4‐fold reductions against BA.4 and BA.5, respectively, compared to the WH‐09 (Figure 2).

**FIGURE 3:**
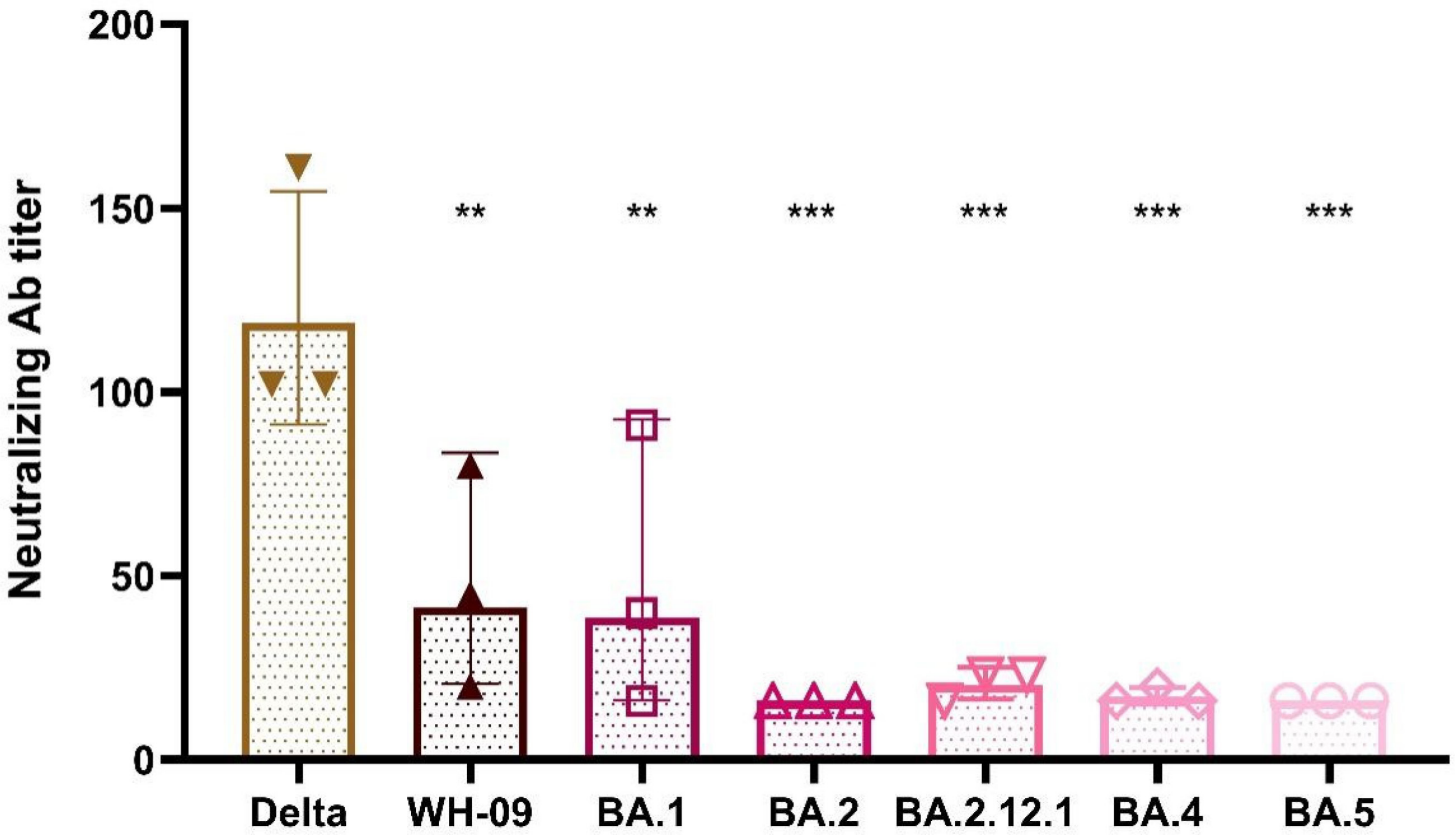
Neutralizing antibody titers against different viruses in sera of macaques after two doses of inactivated vaccine (Delta). The error bars show the standard deviation of three separate experiments.

### 3.3 Cross‐neutralizing antibodies elicited in sera from macaques immunized with two doses of inactivated vaccines (Delta)

We determined neutralizing activity for the WH‐09 variant, Delta variant, and five Omicron subvariants in sera from macaques immunized with two doses of inactivated vaccines (Delta). The sera showed varying degrees of decrease in neutralizing ability against WH‐09 and different Omicron subvariants, with 2.9‐fold and 3.1‐fold reductions in neutralizing activity against WH‐09 and BA.1, but 7.4‐fold, 5.8‐fold, 6.9‐fold, and 7.4‐fold reductions against BA.2, BA.2.12.1, BA.4, and BA.5 compared to the Delta variant (Figure 3).

## 4 DISCUSSION

The peak titer of the Omicron BA.1 subvariant decreased significantly compared with the WH‐09 and Delta variants, while BA.2 and BA.2.12.1 showed an increasing trend relative to BA.1, and BA.4/5 showed the same trend as BA.2 and BA.2.12.1. The trend in the viral loads data was consistent with that of titers. The viral loads of the WH‐09 and Delta strains exhibited similar amplification efficiencies and were higher than Omicron subvariants; similarly, the viral loads of BA.1 were lower than those of the other Omicron subvariants. The reason for the gradual increase in amplification efficiency from BA.1 to BA.4/5 may be that BA.4/5 acquired the L452R mutation, which has been reported to increase SARS‐CoV‐2 fusogenicity and replication in cell culture.^9^, ^10^

Our results in vitro are generally consistent with previous reports. In human lung tissue, the BA.1 subvariant has less severe pathogenicity. In an analogous manner, BA.2 exhibits a low level of pathogenicity in mice and hamsters. However, compared to BA.1, the S protein‐mediated syncytia formation in BA.2 is significantly more effective than it is in BA.1. This may explain why BA.2 has a higher pathogenicity than BA.1.^11^ When compared to the original BA.1 subvariant, the fusogenicity mediated by the S proteins of the BA.2.12.1, BA.4, and BA.5 subvariants is dramatically increased.^12^

Unlike the viral RNA loads continued to increase over 72 h, viral titers only started to decrease after arriving at a peak at 48 or 72 h post infection. Many studies have reported this discrepancy trend between viral loads and titers during SARS‐CoV‐2 replication in Vero E6 cells. This phenomenon may be related to the lack of spike protein on the surface of the virus particles produced at these time points, certain assembly or maturation problems, or virus‐host interactions.^13^, ^14^, ^15^

SARS‐CoV‐2 WH‐09 and Delta vaccines provided good protection against infection with their own virus and part of protection against other mutant strains, albeit accompanied by a decline in neutralizing activity. We also verified that the presence of neutralizing antibodies produced by the two vaccines against Omicron BA.4 and BA.5 subvariants was lower than that of BA.1 and BA.2, consistent with previous studies showing that BA.4 and BA.5 showed stronger immune escape. The immune escape effect of the neutralizing antibodies is related to the mutation of the S protein, including the receptor binding domain (RBD) and the N‐terminal domain, and the mutation of spike protein F486V, which has been shown to have the effect of impairing the neutralizing activity of neutralizing antibodies, has only been founded in BA.4/BA.5, which may be one of the reasons why BA.4/BA.5 had a higher level of immune escape.^16^, ^17^ We also found that the protective effect of Delta vaccines against Omicron BA.4 and BA.5 subvariants was higher than that of the wild‐type vaccine; the reason may be that the mutation L452R, which has been reported to affect vaccine‐induced neutralizing antibodies activity, was possessed by both the Delta variant and BA.4/BA.5, a possibility that needs further verification.

Population‐based studies have shown reduced protection against Omicron subvariants from existing vaccines,^18^ but booster doses were associated with an 86% risk reduction for pneumonia and a 91%–98% risk reduction of developing severe COVID‐19.^19^ The immune barrier established by population vaccination is continuously impacted by the increasing mutation of SARS‐CoV‐2 virus, and improvements in vaccine protection can be achieved by booster vaccination, development of new mutant strain‐specific vaccines, and development of multivalent vaccines.

Our in vitro study of the changes in the replication ability of different Omicron subvariants and the cross‐antibody status of the vaccine against the mutant strains will help us to understand the developmental trends of variation in the virus, assess the harmfulness of the epidemic virus, and provide basic support for research and development of vaccines and drugs. To understand the virulence of the virus, we need to combine the results of animal experiments on virus invasion, distribution, and tissue tropism in vivo, and comprehensively evaluate the trends in changes to the virus.

## AUTHOR CONTRIBUTIONS

Yaqing Zhang, Qi Lv and Linlin Bao conceived and designed the study; Yaqing Zhang, Qi Lv, Fengdi Li, Ran Deng, Xujian Liang, Mingya Liu, and Yiwei Yan performed the experiments; Yaqing Zhang and Feifei Qi collected and analyzed the data; Yaqing Zhang and Qi Lv wrote the original draft of the manuscript; Linlin Bao revised the manuscript. All authors critically read and contributed to the manuscript and approved the final version.

## CONFLICT OF INTEREST

The authors declared no conflict of interest. Linlin Bao is an Editorial Board member of AMEM and a co‐author of this article. To minimize bias, she was excluded from all editorial decision‐making related to the acceptance of this article for publication.
